# Long-Term Outcomes Across Age and Risk Profiles in a Caucasian Living Kidney Donor Cohort

**DOI:** 10.3389/ti.2026.16266

**Published:** 2026-04-23

**Authors:** Claudia Sommerer, Iris Schröter, Nicola Marie Kuhlmann, Zoi Bougioukou, Martin Zeier

**Affiliations:** 1 Department of Nephrology, University Hospital Heidelberg, Heidelberg, Germany; 2 German Centre for Infection Research (DZIF), Site Heidelberg, University Heidelberg, Heidelberg, Germany

**Keywords:** age, cardiovascular risk, living kidney donation, renal function, risk score

## Abstract

Living kidney donation achieves excellent recipient outcomes, but increasingly involves older and medically complex donors, while long-term data across age groups remain limited. The Heidelberg Kidney Donor Study followed 632 donors (1991–2020), stratified by age <40 (n = 93), 40–60 (n = 424), and >60 years (n = 115). Primary outcomes were a ≥50% eGFR decline and an eGFR <45 mL/min/1.73 m^2^ at long-term follow-up. Early post-donation adaptation, long-term eGFR trajectories, cardiovascular events, and risk patterns were evaluated. Mean donor age was 50.6 ± 10.6 years (62.5% female). eGFR declined by 26.0% after donation and remained stable thereafter. At a median follow-up of 12 years, ≥50% eGFR decline occurred in 4.8%, 5.3%, and 14.4% of donors aged <40, 40–60, and >60 years, respectively, an eGFR <45 mL/min/1.73 m^2^ in 1.2%, 5.3%, and 20.4%. An eGFR <30 mL/min/1.73 m^2^ occurred in 1.2%, major adverse cardiovascular events in 4.3%. Age, hypertension, and baseline-eGFR independently predicted renal impairment. Younger donors with hypertension or obesity had up to a 14.3% risk of ≥50% eGFR decline, exceeding the risk in healthy older donors (12.5%). Living kidney donation was associated with stable long-term kidney function after early adaptation, with substantial heterogeneity driven more by baseline renal reserve and comorbidity than chronological age alone.

## Graphical Abstract

**Figure:**
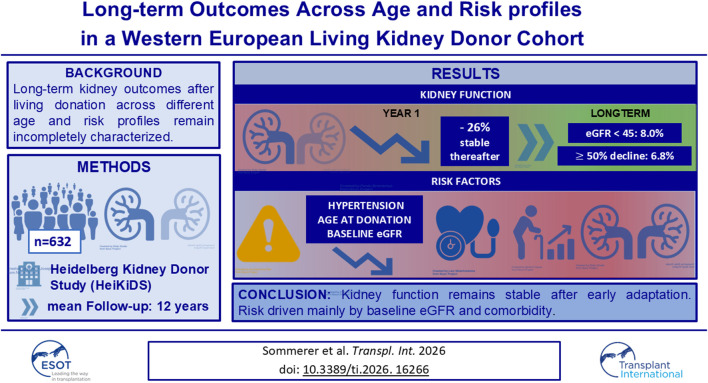

## Introduction

Living kidney donation (LKD) is the optimal treatment for renal failure, increasing organ availability, shortening waiting times and enabling preemptive transplantation with superior graft and patient survival [1–3]. Most international and national cohort studies have demonstrated no significant long-term harm to donors [4–9]. However, two more recent studies identified a modestly higher risk of endstage renal disease (ESRD) in living kidney donors compared with the general population [10, 11], providing limited data on outcomes for older donors and those with cardiometabolic comorbidities.

Due to organ shortages and long waiting times, transplant centers increasingly accept medically complex living kidney donors – older individuals or those with hypertension and obesity [12], consistent with recent registry observations [13]. This shift may increase donors’ risk for renal and cardiovascular events and affect transplant ouctomes [12, 14]. Such donors now account for 25%–50% of LKD program [15]. Acceptance criteria vary widely between transplant centers [16].

Despite the importance of this issue, high-quality studies with long-term outcomes are scarce [9, 14, 17, 18]. Most available data derive from retrospective registry-based analyses focusing on hard endpoints, with limited insight into longitudinal kidney function trajectories or clinically relevant heterogeneity among donors.

The aim of the present study was to characterize long-term renal and cardiovascular outcomes after living kidney donation across donor age groups using detailed longitudinal follow-up data. A central objective was to disentangle whether advanced donor age predominantly affects the level of kidney function achieved after donation or whether it is associated with an accelerated rate of subsequent kidney function decline over time.

In addition, the study aimed to assess the modifying role of baseline renal reserve and common comorbidities and to describe heterogeneity in long-term renal risk based on routinely collected donor characteristics.

## Materials and Methods

### Study Design

The Heidelberg Kidney Donor Study (HeiKiD) is a prospective cohort study established to evaluate long-term outcomes after LKD and to enhance shared decision-making and informed consent.

The study was approved by the ethics committee of the University Hospital Heidelberg (S104-2011), and all participants provided written informed consent. Data collected prior to 2011 were obtained retrospectively from medical records. Since 2011, donors have been followed prospectively within a structured cohort study with predefined follow-up intervals.

Data handling complied with the European General Data Protection Regulation.

Eligible donors were aged ≥18 years, had completed the pre-donation evaluation, and had at least 5 years of follow-up after donation. The analysis included 632 caucasian donors who donated between 01/1991 and 06/2020 for recipients across 48 dialysis centers in southern and central Germany. Demographic data were obtained via structured questionnaires. Clinical and laboratory data were collected before donation, 1 year post-donation, and annually thereafter. In accordance with German living donor evaluation standards, all potential donors underwent comprehensive cardiovascular assessment before donation, including echocardiography and exercise ECG; if coronary heart disease was known or non-invasive testing was abnormal, coronary angiography was performed. Donors with clinically significant or uncontrolled cardiovascular disease were generally excluded, and only mild, stable, or previously treated cardiovascular conditions were accepted after interdisciplinary evaluation.

### Study Outcomes

Primary outcomes were:

- ≥50% decline in estimated glomerular filtration rate (eGFR) from baseline to last follow-up
- prevalence of eGFR <45 mL/min/1.73 m^2^ at long-term follow-up.

The threshold corresponds to CKD stage 3b and was selected because this level of kidney function has been consistently associated with a substantially increased risk of cardiovascular events, CKD progression, and mortality in large population-based studies [19, 20].

Secondary endpoints included the incidence of eGFR <30 mL/min/1.73 m^2^, the need for renal replacement therapy, composite major adverse cardiovascular events (MACE), individual components of MACE (myocardial infarction, stroke, cardiovascular death, peripheral artery disease events (revascularization, amputation)), and death.

Beyond binary renal endpoints, analyses focused on early post-donation change, achieved post-adaptation kidney function, and long-term eGFR trajectories.

Analyses were stratified by donor age (<40 years, 40–60 years, and >60 years) to explore age effects. In addition, the impact of baseline comorbidity on longterm outcome was analyzed. A dedicated subanalysis was performed in donors aged ≥70 years.

### Statistical Methods

Continuous variables were expressed as mean ± standard deviation (SD) or median (interquartile range, IQR) and compared using the Student’s t-test or Mann–Whitney U test. Categorical variables were presented as counts and percentages and compared using the chi-square or Fisher’s exact test. Bias was minimized through consecutive enrollment and consistent data collection. Analyses were performed using complete case data without imputation.

Linear mixed-effects models were used to evaluate the association of age at donation with eGFR levels and longitudinal eGFR trajectories.

To separate the expected early post-nephrectomy adaptation from subsequent long-term changes, early eGFR change was quantified from baseline to 12 months after donation. Early change was defined as ΔeGFR0–12 (eGFR1y − eGFRbaseline) and, in sensitivity analyses, as percent change (%ΔeGFR). Determinants of early eGFR change were assessed using multivariable linear regression with prespecified predictors: baseline eGFR (CKD-EPI), age at donation (continuous), preexisting hypertension, BMI, sex, and donation era (grouped into 1991–2000, 2001–2010, 2011–2020). To facilitate comparison of effect sizes across predictors, standardized regression coefficients were calculated for continuous variables.

To assess determinants of achieved kidney function at long-term follow-up, multivariable linear regression models were fitted with eGFR at last follow-up as the dependent variable and adjusted for baseline eGFR, early eGFR change (ΔeGFR0–12), follow-up duration, age at donation, hypertension, BMI, and sex.

#### Risk Grouping and Regression Analysis

Donors were classified by age and presence of established risk factors for kidney function decline. Associations between baseline variables and outcomes were assessed using univariate logistic regression. Multivariate models included prespecified clinically relevant variables.

#### Exploratory Risk Stratification Using Baseline Clinical Variables

An exploratory composite score was constructed to illustrate the combined association of key baseline factors with long-term outcomes. Independent predictors from the multivariate analysis were used in an exploratory manner to summarize their joint association with long-term renal outcomes. Regression coefficients (β) were used to assign relative weights to each predictor, reflecting their contribution within this cohort. An individual composite score was calculated as the sum of weighted predictors. For descriptive purposes, risk scores were categorized into tertiles representing low, intermediate, and high-risk groups, and the observed incidence of both outcomes was calculated for each category. Model calibration was assessed exploratorily by comparing predicted and observed outcome frequencies across tertiles and deciles, including calibration plots, calibration slope and intercept, and the Hosmer–Lemeshow test. Discriminative performance was quantified using the area under the receiver operating characteristic curve (AUC).

All analyses were performed in R (Version 2024.12.0).

## Results

### Cohort Characteristics

A total of 632 living kidney donors with a minimum follow-up of 5 years were included. Of these, 93 were aged <40 years (14.7%), 424 were aged 40–60 years (67.1%), and 115 were aged >60 years (16.6%), Table 1. The mean age at donation was 50.6 ± 10.6 years (range 19–77). Mean donor age increased from 43.9 years in 1990–1994 to 53.4 years in 2015–2020.

**TABLE 1:** Baseline Characteristics of the Living Kidney Donor Cohort and Median Follow-Up, stratified by Age.

Parameter	n	Total cohort	<40 years	40–60 years	>60 years	p-value
n	​	632	93	424	115	​
Demographics						
Age (mean ± SD)	632	50.6 ± 10.6	33.3 ± 5.3	50.3 ± 5.6	65.5 ± 3.7	**<0.001**
Male gender (%)	632	37.5 (237)	29.0 (27)	37.0 (157)	46.1 (53)	**0.0387**
BMI (kg/m^2^)	615	26.2 ± 4.1	25.4 ± 4.8	26.4 ± 4.0	26.3 ± 3.6	**0.0405**
18-25 kg/m^2^	​	41.1 (253)	49.4 (44)	40.6 (168)	36.6 (41)	​
<18 kg/m^2^	​	1.0 (6)	3.4 (3)	3.1 (13)	​	​
>25 kg/m^2^	​	40.5 (249)	27.0 (24)	43.2 (175)	44.6 (50)	​
Obesity class I (BMI 30-35 kg/m^2^)	​	14.1 (87)	15.7 (14)	13.3 (55)	16.1 (18)	​
Obesity class II (BMI 35-40 kg/m^2^)	​	2.8 (17)	3.4 (3)	3.1 (13)	0.9 (1)	​
Obesity class III (BMI >40 kg/m^2^)	​	0.5 (3)	1.1 (1)	0.5 (2)	1.8 (2)	​
Nicotine use (active smoker)	603	24.2 (146)	31.4 (27)	26.6 (107)	10.5 (12)	**0.0008**
Donated to	630	​	​	​	​	​
Related	​	63.7 (392)	80.0 (72)	61.7 (259)	58.4 (66)	**0.0012**
First-degree relatives	​	61.1 (385)	73.1 (68)	60.0 (253)	55.7 (64)	**0.0150**
Unrelated	​	36.2 (226)	20.0 (18)	38.3 (161)	51.6 (47)	**0.0012**
Spouse	​	31.6 (200)	17.2 (16)	34.0 (144)	34.8 (40)	**0.0051**
Sibling	​	13. 9 (88)	29.0 (27)	12.3 (52)	7.8 (9)	**<0.001**
Mother -- child	​	29.3 (185)	35.5 (33)	28.5 (121)	27.0 (31)	0.3427
Father -- child	​	18.2 (115)	9.6 (8)	19.3 (82)	21.7 (25)	**0.0288**
Friend	​	1.9 (12)	1.1 (1)	2.6 (11)	5.2 (6)	0.4978
Blood pressure parameters						
Mean arterial pressure (mmHg)	618	96.8 ± 9.8	93,2 ± 9.3	96.6 ± 9.4	99.9 ± 10.4	**<0.001**
Systolic BP (mmHg)	618	128.5 ± 15.1	123.2 ± 13.2	127.9 ± 14.6	134.9 ± 16.2	**<0.001**
Diastolic BP (mmHg)	618	80.8 ± 8.5	78.3 ± 8.6	81.0 ± 8.2	82.5 ± 9.2	**0.0097**
24h systolic BP mean (mmHg)	467	125.6 ± 13.0	121.9 ± 13.5	125.2 ± 13.1	130.4 ± 10.6	**<0.001**
24h diastolic BP mean (mmHg)	466	77.4 ± 8.9	74.5 ± 7.2	77.7 ± 9.2	78.7 ± 8.5	**0.0054**
Medical history						​
Hypertension	632	44.5 (281)	24.7 (23)	43.2 (183)	65.2 (75)	**<0.001**
Diabetes mellitus	632	0.0 (0)	0.0 (0)	0.0 (0)	0.0(0)	-
Prediabetes	595	5.2 (31)	3.4 (3)	4.8 (19)	8.1 (9)	0.2691
Hyperlipidemia	632	8.6 (54)	2.2 (2)	7.8 (33)	16.5 (19)	**<0.001**
Metabolic syndrome	611	7.7 (49)	10.2 (9)	7.5 (31)	6.3 (7)	0.5624
Thyroid disease	632	17.3 (109)	5.4 (5)	18.0 (76)	24.0 (26)	**0.001**
Cardiovascular disease I	632	0.9 (6)	0.0 (0)	0.7 (3)	2.6 (3)	0.15
Malignant diseases	632	2.2 (14)	0.0 (0)	2.4 (10)	2.6 (3)	0.400
COPD/Asthma/Chronic bronchitis	632	3.8 (24)	2.2 (2)	4.3 (18)	3.6 (4)	0.6256
Urological diseases	632	4.6 (29)	1.1 (1)	4.5 (19)	7.8 (9)	0.062
Gynecological diseases	632	6.3 (40)	5.4 (4)	7.8 (33)	1.7 (2)	0.057
Psychiatric disorders	632	2.5 (16)	0.0 (0)	2.8 (12)	3.5 (4)	0.4181
Family medical history						
Kidney disease	388	24.5 (95)	32.5 (13)	21.1 (58)	32.9 (24)	0.0528
Medication use						
Antihypertensive medication	632	24.4 (154)	3.2 (3)	23.1 (98)	46.1 (53)	**<0.001**
Number of antihypertensives	632	0.36 ± 0.74	0.04 ± 0.25	0.33 ± 0.67	0.76 ± 1.03	**<0.001**
Lipid-lowering drugs	632	5.1 (32)	0.0 (0)	4.0 (17)	13.0 (15)	**<0.001**
Thyroid medication	632	12.5 (79)	1.1 (1)	13.4 (57)	18.3 (21)	**0.0006**
Pain medication (non-NSAIDs)	632	0.2 (1)	0.0 (0)	0.2 (1)	0.0 (0)	0.7822
Antidepressants	632	2.1 (13)	0.0 (0)	2.4 (10)	2.6 (3)	0.3138
Kidney data						
Kidney length right (mm) (mean ± SD)	575	112.0 ± 9.2	112.5 ± 13.6	112.4 ± 8.2	110.5 ± 8.5	**0.041**
Kidney length left (mm) (Mean ± SD)	574	112.6 ± 9.6	112.2 ± 14.3	113.1 ± 8.7	111.3 ± 8.2	**0.0049**
MAG3-clearance (mL/min/1.73m^2^)	513	232.2 ± 44.4	254.9 ± 43.0	232.9 ± 44.2	212.6 ± 37.1	**<0.001**
Renal function right side (%, mean ± DS)	559	0.50 ± 0.04	0.50 ± 0.04	0.50 ± 0.04	0.50 ± 0.04	0.5434
Renal function left side (%, mean ± SD)	559	0.50 ± 0.04	0.50 ± 0.04	0.50 ± 0.04	0.50 ± 0.04	0.5282
Renal function donated kidney (%, mean ± SD)	559	0.49 ± 0.04	0.48 ± 0.04	0.49 ± 0.04	0.49 ± 0.04	0.8883
Hematuria	628	9.6 (60)	4.3 (4)	11.2 (47)	7.9 (9)	0.1005
Proteinuria^a^ (g/L) (Median IQR)	613	0.037 (0.018–0.065)	0.034 (0.013–0.067)	0.037 (0.0195–0.0645)	0.043 (0.020–0.062)	0.626
Proteinuria^a^ >0.15 (g/L)	613	2.9 (18)	5.6 (5)	2.9 (12)	0.91 (1)	0.1408
Pre-donation biopsy	631	2.2 (14)	0.0 (0)	2.1 (9)	4.3 (5)	0.1039
Donated kidney left side	598	53.0 (318)	46.0 (55)	53.0 (216)	50.0 (56)	0.8000
Median follow-up years (Md, IQR)	632	12.3 (8.8–16.2)	13.9 (10.0–18.6)	12.1 (8.6–16.2)	11.8 (8.6–14.9)	**0.0046**

Continuous variables are presented as mean ± standard deviation (SD), and group comparisons were performed using the non-parametric Kruskal-Wallis test. Categorical variables are shown as percentages with the absolute number in parentheses, and were compared using the Chi-square test or Fisher’s exact test, as appropriate. A p-value less than 0.05 was considered statistically significant.

Abbreviations: BMI -Body Mass Index; Obesity class I: BMI, 30–34.9 kg/m^2^; Obesity class II: BMI, 35–39.9 kg/m^2^; Obesity class III: BMI ≥40 kg/m^2^. BP: blood pressure; SPB: COPD: chronic obstructive pulmonary disease; MAG3: Mercaptoacetyltriglycine (renal scintigraphy agent); NSAIDs: Non-Steroidal Anti-Inflammatory Drugs; n–number; SD: standard deviation.

Bold values indicate statistical significance.

^a^ Measured in spot urine samples.

Overall, 62.5% were female, ranging from 53.9% among donors >60 years of age to 71.0% in the youngest group. Nearly two-thirds were related to their recipients (392/632; 62.0%), most frequently parent-to-child, especially mother-to-son (16.9%), Table 1.

The median follow-up time after donation was 12 years (IQR 9–16).

### Cardiometabolic History and Baseline Renal Findings

Cardiometabolic profiles showed clear age-related trends. Mean BMI was comparable across all groups, but overweight and obesity were more common in older donors. Smoking was more prevalent in younger individuals, while hypertension and the use of antihypertensives increased with age. Accordingly, office and 24-h blood pressure measurements were higher in older donors. Dyslipidemia and thyroid disorders also increased with age (Table 1).

Hematuria was observed in 9.6% of donors and occurred more frequently in female than male donors (12.8% vs. 4.2%). In cases of unexplained hematuria, a pre-donation kidney biopsy was performed in 14 donors. Histopathological findings were generally mild and major glomerulopathies were excluded. Thin basement membrane alterations were reported in 3 donors.

### History of Malignancy and Psychiatric Disorders

**FIGURE 1:**
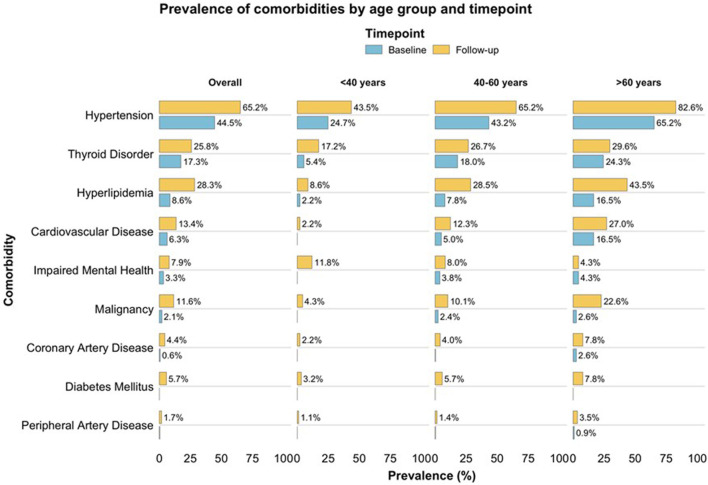
Prevalence of comorbidities at baseline and at long-term follow-up after living kidney donation. Stacked horizontal bar plots show the percentage of donors with selected comorbidities before donation (blue) and at last follow-up (yellow), presented overall and by age group (<40, 40–60, >60 years).

A history of malignancy was documented in 14 donors (2.2%), including breast cancer (n = 2), cervical cancer (n = 3), colon cancer (n = 1), papillary thyroid carcinoma (n = 1), renal cell carcinoma (n = 1), seminoma (n = 1), appendiceal carcinoid tumor (n = 1), pleural tumor (n = 1), and basal cell carcinoma (n = 3). Psychiatric disorders were reported in 16 donors (2.5%), predominantly depressive disorders (n = 12), followed by bipolar disorder (n = 2), panic attacks (n = 1), and adjustment disorder/burnout (n = 1). The prevalence of these comorbidities at baseline and at long-term follow-up is shown in Figure 1, both overall and stratified by age group (<40, 40–60, >60 years).

### Descriptive Course of Kidney Function

Baseline-eGFR decreased progressively with age and fell by 0.58 mL/min/1.73 m^2^ per year of age (β = −0.58; 95% CI −0.67 to −0.48; p = 3.26 × 10^−2^), which corresponds to −5.76 mL/min/1.73 m^2^ per decade.

**FIGURE 2:**
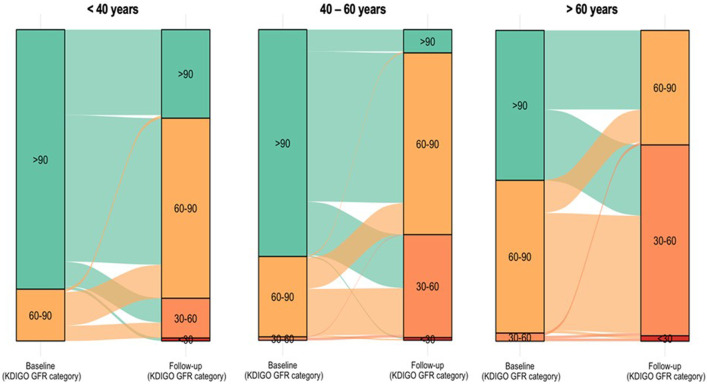
Transition of CKD stages before donation and at long-term follow-up in donors <40 years, 40-60 years and >60 years. Alluvial plots show transitions of donors between KDIGO eGFR categories (>90, 60–90, 45–60, 30–45, <30 mL/min/1.73 m^2^) from baseline (left) to last follow-up (right) in three age groups: <40 years, 40–60 years, and >60 years. The width of each flow represents the number of donors moving between categories.

**FIGURE 3:**
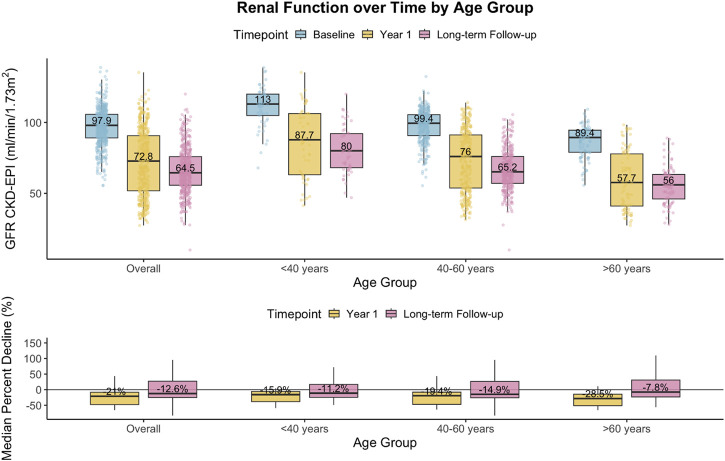
Changes in estimated glomerular filtration rate (eGFR, CKDepi) after living kidney donation. Top panel: Boxplots show eGFR (mL/min/1.73 m^2^) at baseline (blue), 1 year after donation (yellow), and long-term follow-up (pink) overall and by donor age group (<40, 40–60, >60 years). Numbers within boxes indicate group medians. Bottom panel: Median percent decline in eGFR relative to baseline (year 1) and relative to year 1 at long-term follow-up. Values represent median (interquartile range). The greatest early decline occurred in donors >60 years (−28% at year 1), with minimal further change thereafter.

During the study, CKDepi eGFR decreased from 95.8 ± 14.3 to 66.5 ± 16.6 mL/min/1.73 m^2^ (Δ-30.6%, p < 0.001, Table 2). This decline was observed in all age groups but was most pronounced in donors >60 years (−35.1%, p < 0.001). Transitions between eGFR categories are shown in Figure 2. Within the first year after donation, eGFR decreased by an average of 26.0% (Δ–22.4% < 40 years; 24.9% 40–60 years; −31.8% > 60 years) and remained stable with minimal long-term change (+0.4%, −1.2%, +3.2%, respectively), Figure 3.

**TABLE 2:** Longitudinal Changes in Laboratory Parameters, stratified by Age.

Laboratory Values	Group	Baseline (mean ± SD)	Last follow-up (mean ± SD)	p-value (longitudinal)	p-value (age groups)
CKDepi GFR (mL/min/1.73m^2^)	Total	95.8 ± 14.3	66.5 ± 16.6	<0.001	**<0.001**
	<40	104.9 ± 19.3	77.9 ± 19.5	<0.001	​
	40–60	96.3 ± 12.6	66.7 ± 15.0	<0.001	​
	>60	86.7 ± 11.0	56.3 ± 13.3	<0.001	​
MDRD GFR (mL/min/1.73m^2^)	Total	100.6 ± 20.7	62.3 ± 15.2	<0.001	**<0.001**
	<40	107.0 ± 27.0	69.8 ± 15.5	<0.001	​
	40–60	100.5 ± 19.2	61.9 ± 14.4	<0.001	​
	>60	95.8 ± 18.6	57.4 ± 15.5	<0.001	​
Creatinine (mg/dL)	Total	0.78 ± 0.16	1.07 ± 0.32	<0.001	**0.001**
	<40	0.79 ± 0.19	0.99 ± 0.22	<0.001	​
	40–60	0.78 ± 0.15	1.06 ± 0.33	<0.001	​
	>60	0.79 ± 0.16	1.14 ± 0.30	<0.001	​
Cholesterol (mg/dL)	Total	203 ± 41	208 ± 38	0.287	​
	<40	202 ± 42	191 ± 39	0.0584	​
	40–60	206 ± 40	210 ± 36	0.746	​
	>60	196 ± 44	210 ± 40	0.00168	​
LDL cholesterol (mg/dL)	Total	126 ± 32	120 ± 35	0.0084	0.616
	<40	118 ± 37	123 ± 34	0.0614	​
	40–60	127 ± 31	120 ± 35	0.0668	​
	>60	126 ± 32	115 ± 36	0.0005	​
HDL cholesterol (mg/dL)	Total	59 ± 16	59 ± 17	0.1502	0.093
	<40	55 ± 17	57 ± 17	0.7374	​
	40–60	60 ± 17	59 ± 18	0.0459	​
	>60	59 ± 15	61 ± 16	0.5115	​
Triglycerides (mg/dL)	Total	114 ± 73	139 ± 123	<0.001	0.370
	<40	101 ± 73	129 ± 81	0.0066	​
	40–60	117 ± 76	144 ± 134	<0.001	​
	>60	110 ± 58	130 ± 104	0.0826	​
TSH (mU/L)	Total	1.48 ± 1.44	1.82 ± 1.20	<0.001	0.891
	<40	1.84 ± 1.18	2.06 ± 1.59	0.0982	​
	40–60	1.45 ± 1.58	1.73 ± 0.97	<0.001	​
	>60	1.33 ± 0.97	2.00 ± 1.57	0.0016	​
Hemoglobin (g/dL)	Total	14.07 ± 1.29	14.02 ± 1.51	0.7636	0.139
	<40	13.86 ± 1.41	13.97 ± 1.74	0.2376	​
	40–60	14.08 ± 1.30	14.11 ± 1.46	0.1775	​
	>60	14.15 ± 1.10	13.70 ± 1.49	0.0092	​
HbA1c (%)	Total	5.45 ± 0.40	5.56 ± 0.58	<0.001	0.440
	<40	5.34 ± 0.37	5.36 ± 0.55	0.301	​
	40–60	5.42 ± 0.38	5.59 ± 0.64	<0.001	​
	>60	5.62 ± 0.43	5.57 ± 0.58	0.905	​
Urea (mg/dL)	Total	27.8 ± 7.6	35.2 ± 13.6	<0.001	**<0.001**
	<40	26.2 ± 7.5	30.3 ± 9.7	<0.001	​
	40–60	27.8 ± 7.8	34.9 ± 12.9	<0.001	​
	>60	29.3 ± 6.5	39.9 ± 16.9	<0.001	​
Phosphate (mg/dL)	Total	1.03 ± 0.23	1.06 ± 0.32	0.102	0.230
	<40	1.05 ± 0.18	1.01 ± 0.32	0.265	​
	40–60	1.03 ± 0.24	1.06 ± 0.30	0.0304	​
	>60	1.02 ± 0.21	1.09 ± 0.41	0.563	​

Mean ± SD, values represent the average laboratory measurements and their Standard deviation (SD) for each parameter, stratified by age group and timepoint (baseline before donation and long-term follow-up. Longitudinal comparisons between T0 and LFUP, within each age group were performed using paired Wilcoxon signed-rank tests to assess whether changes over time are statistically significant. Between-group comparisons at the LFUP, timepoint were conducted using.

Kruskal-Wallis tests to evaluate differences across age groups. P-values are reported for both the longitudinal and between-group (age group) tests. A p-value less than 0.05 was considered statistically significant.

Abbreviations: Hb–hemoglobin; Hct–hematocrit; MDRD GFR, glomerular filtration rate estimated by Modification of Diet in Renal Disease equation; CKDepi GFR, glomerular filtration rate estimated by Chronic Kidney Disease Epidemiology Collaboration equation; HbA1c–glycated hemoglobin A1c; HDL, high-density lipoprotein; LDL, low-density lipoprotein; n - number; iPTH, intact parathyroid hormone; TSH, thyroid-stimulating hormone.

Bold values indicate statistical significance.

**FIGURE 4:**
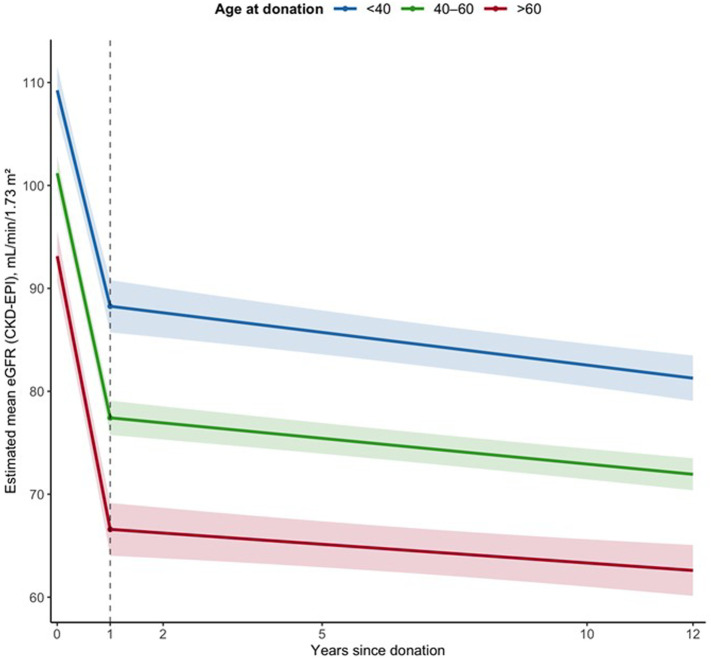
Change in eGFR from baseline after living kidney donation according to cardiometabolic risk profile and age. Mean ΔeGFR from baseline at 1 year and long-term follow-up is shown for donors with no risk factors, hypertension, obesity, or both obesity and hypertension, stratified by age group (<40, 40–60, >60 years). All groups demonstrate the expected early decline in eGFR after donation followed by relative stabilization. Error bars indicate 95% confidence intervals.

Across longitudinal follow-up, older age at donation was associated with lower eGFR values (−0.65 mL/min/1.73 m^2^ per year of age, p < 0.001), whereas the rate of eGFR decline did not differ by age group (interaction p = 0.54). Donors with hypertension demonstrated the largest early decline and slightly lower long-term eGFR values (Figure 4).

### ≥ 50% Decline in eGFR at Long-Term Follow-Up and Prevalence of eGFR <45 mL/min/1.73 m^2^

**FIGURE 5:**
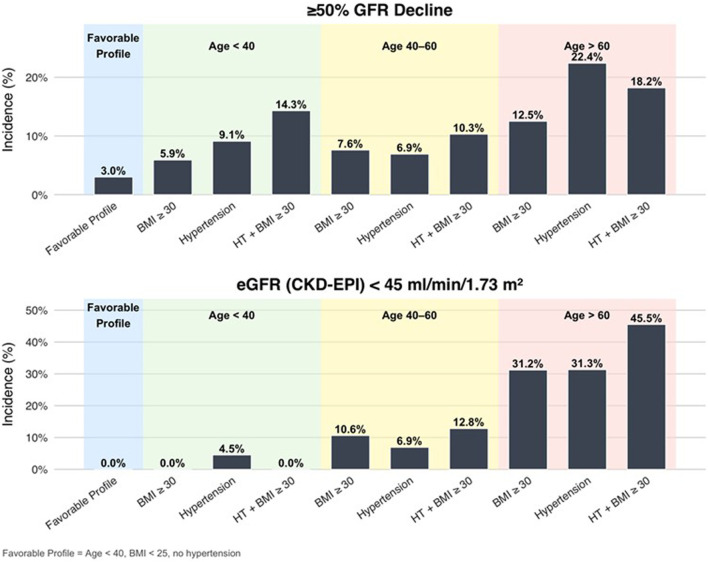
Incidence of adverse renal outcomes by age group and baseline risk profile. Bar plots show the percentage of donors who developed (top) ≥ 50% eGFR decline from baseline and (bottom) an eGFR <45 mL/min/1.73 m^2^ at long-term follow-up. Results are stratified by age group (<40, 40–60, >60 years) and baseline risk profile. “Favorable profile” = age <40 years, BMI <25 kg/m^2^, and no hypertension. The combination of older age (>60 years), baseline hypertension, and BMI ≥30 kg/m^2^ was associated with the highest incidence of both outcomes, reaching 22.4% for ≥50% eGFR decline and 45.5% for eGFR <45 mL/min/1.73 m^2^.

A ≥50% eGFR decline at long-term was detected in 6.8% of donors, with the prevalence increasing sharply with age (4.8% < 40 years, 5.3% 40–60 years, 14.4% > 60 years, p = 0.0053), Table 3. Even among younger donors, the incidence ranged from 3.0% in donors with a favorable profile to 14.3% in donors with both hypertension and obesity. In older donors, the risk was 12.5% in donors without risk factors, 22.4% in donors with one risk factor, and 45.5% in hypertensive obese donors (Figure 5).

**TABLE 3:** Long-Term Outcome after Living Kidney Donation, stratified by Age.

Outcome	Total cohort	<40 years	40–60 years	>60 years	p-value
≥50% eGFR decline	6.8 (40)	4.8 (4)	5.3 (21)	14.4 (15)	**0.0053** ^a^
eGFR <45 mL/min/1.73 m^2^	8.0 (47)	1.0 (1)	5.5 (21)	20.4 (25)	**<0.001**
eGFR <30 mL/min/1.73 m^2^	1.2 (7)	1.2 (1)	1.0 (4)	1.9 (2)	0.6090^a^
Renal replacement therapy	0.2 (2)	0.1 (1)	0.2 (1)	0.0 (0)	—
MACE (composite)	4.3 (27)	2.2 (2)	4.0 (17)	7.0 (8)	0.2300^b^
Myocardial infarction	0.6 (4)	0.1 (1)	0.7 (3)	0.0 (0)	0.3968
Stroke	1.7 (11)	0.0 (0)	1.2 (6)	4.3 (5)	**0.0432**
Cardiovascular death	0.3 (2)	0.0 (0)	0.2 (1)	0.9 (1)	0.4603
Peripheral artery disease events	1.4 (9)	1.2 (1)	0.7 (5)	1.7 (3)	0.7045
Death	3.3 (21)	0.0 (0)	2.6 (11)	8.7 (10)	**0.0012** ^b^

^a^ Trend p-value (Cochran–Armitage).

^b^ Group p-value (chi-square/Fisher, as appropriate).

eGFR, estimated glomerular filtration rate; MACE, major adverse cardiac event.

Bold values indicate statistical significance.

eGFR <45 mL/min/1.73 m^2^ was identified in 8.0% of donors, with the prevalence increasing sharply with age (1.2% < 40 years, 5.3% 40–60 years, 20.4% > 60 years). The incidence of an eGFR <45 mL/min/1.73 m^2^ rose further with the presence of risk factors such as hypertension and obesity. In older donors, the risk was 12.5% in donors without risk factors, 22.4% in donors with one risk factor, and 45.5% in hypertensive obese donors (Figure 5).

### Factors Associated With a ≥50% Decline in eGFR at Long-Term Follow-Up

In the univariate analysis (Table 4), age at donation strongly predicted a ≥50% decline in eGFR: each additional year increased the probability (≈odds) of a substantial GFR loss by about 4.7% (OR 1.047, p = 0.006). Donors >60 years had a more than three times higher risk compared to donors <40 years (OR 3.34, p = 0.038).

**TABLE 4:** Univariate Logistic Regression Analysis of Predictors for eGFR Decline ≥50% at Long-Term Follow-Up.

Characteristics	OR	95%-CI	p
Demographics			
Age (years) at donation	1.047	1.013; 1.082	**0.006**
<40 years	Reference	​	​
40-60 years	1.159	0.388:3.460	0.791
>60 years	3.337	1.068; 10.427	**0.038**
Male gender	1.043	0.583; 2.163	0.736
BMI (kg/m^2^)	1.016	0.940; 1.099	0.684
BMI >30 kg/m^2^	1.243	0.555; 2.786	0.597
Active smoker	1.046	0.480; 2.279	0.909
Blood pressure parameters			
Mean arterial pressure (mmHg)	1.040	1.009; 1.072	**0.012**
Systolic BP (mmHg)	1.030	1.010; 1.050	**0.003**
Diastolic BP (mmHg)	1.029	0.992; 1.067	0.124
24h systolic BP (mmHg)	1.035	1.009; 1.062	**0.009**
24h diastolic BP (mmHg)	1.035	0.992; 1.081	**0.112**
History of comorbidities			
Hypertension	3.557	1.744; 7.256	**<0.001**
Prediabetes	1.075	0.246; 4.696	0.923
Hyperlipidemia	1.580	0.592; 4.217	0.361
Metabolic syndrome	0.935	0.219; 2.730	0.931
Thyroid disease	2.910	1.419; 5.965	**0.004**
Cardiovascular disease	3.010	0.34; 26.36	0.321
Malignant diseases	1.14	0.15; 8.94	0.901
COPD/Asthma/Chronic bronchitis	1.344	0.305–5,932	0.696
Medication use			
Antihypertensive medication	2.19	1.133; 4.246	**0.020**
Number of antihypertensives	1.390	0.981; 1.969	**0.064**
Lipid-lowering drugs	2.99	1.085; 8.236	**0.034**
Thyroid medication	2.527	1.183; 5.394	**0.017**
Baseline laboratory			
Creatinine (mg/dL)	0.730	0.095; 5.583	0.761
MDRD eGFR (mL/min/1.73 m^2^)	0.997	0.981; 1.012	0.666
CKDepi eGFR (mL/min/1.73 m^2^)	0.991	0.970; 1.013	0.433
Cockroft-gault-eGFR (mL/min)	0.991	0.979; 1.003	0.168
MAG3-clearance (mL/min/1.73m^2^)	0.996	0.988; 1.004	**0.302**
Creatinine-clearance (mL/min/1.73m^2^)	0.989	0.976; 1.002	**0.094**
Hemoglobin (g/dL)	1.089	0.846; 1.402	0.509
Cholesterol (mg/dL)	0.995	0.986; 1.004	0.256
Triglycerids (mg/dL)	0.999	0.993; 1.004	0.680
LDL cholesterol (mg/dL)	0.995	0.982; 1.007	0.413
HDL cholesterol (mg/dL)	0.992	0.967; 1.018	0.539
Blood glucose (mg/dL)	1.000	0.979; 1.023	0.968
HbA1c (%)	1.312	0.517; 3.376	0.561
TSH (µIU/mL)	1.170	0.997; 1.374	0.055
Phosphate	1.399	0.370; 5.291	0.806
iPTH	1.011	0.652; 1.567	0.962
Urea (mg/dL)	0.994	0.951; 1.040	0.806
Urine albumine (mg/L)	0.983	0.863; 1.121	0.803
Urine protein (g/L)			
<0.03 g/L	Reference	​	​
0,03-0.15 g/L	1.008	0.518; 1.986	0.981
>0.15 g/L	3.348	0.872; 12.858	0.078
Long-term follow-up data			
Time since donation (months)	1.006	1.002–1.011	**0.002**
Age at follow-up (years)	1.075	1.040; 1.112	**<0.001**
Comorbidities			
Hypertension	2.136	1.001; 4.560	**0.049**
Diabetes mellitus	9.200	0.213; 3.964	0.910
Hyperlipidemia	1.693	0.872; 3.286	**0.120**
Thyroid disease	6.280	0.273; 1.445	0.273
Cardiovascular disease	2.320	0.927; 5.806	0.072
Malignant diseases	2.391	1.095; 5.223	**0.029**
Major cardiovascular events	-	0.00-lnf	0.984

Bold values indicate statistical significance.

Among preexisting comorbidities, hypertension and thyroid disease proved to be significant predictors, while hyperlipidemia, cardiovascular disease, and prediabetes were not predictive. The use of antihypertensive, lipid-lowering, and thyroid medications were also linked to a higher risk.

Each additional month since donation, increased the risk. Older age at follow-up also remained a strong predictor. At long-term follow-up, hypertension (both preexisting and new-onset) was associated with GFR loss, and the presence of malignancy also increased the risk.

### Factors Associated With an eGFR <45 mL/min/m^2^ at Long-Term Follow-Up

Older age at donation increased the long-term risk of an eGFR <45 mL/min/1.73 m^2^ by approximately 10.6% per years (OR 1.11, p < 0.001), Table 5. Donors older than 60 years had a substantially higher risk compared with those younger than 40 years (OR 6.62, p < 0.001). Pre-existing hypertension was associated with an approximately twofold increased risk of an eGFR <45 mL/min/1.73 m^2^ (OR 2.07, p = 0.002. A similar increase was observed for hyperlipidemia (OR 2.40, p = 0.036). Thyroid disease was also associated with a significantly higher risk of reduced eGFR (OR 2.30, p = 0.020).

**TABLE 5:** Univariate Logistic Regression Analysis: eGFR CKDepi <45 mL/min/1.73 m2 at Long-Term Follow-Up.

Characteristics	OR	95%-CI	p
Demographics			
Age (years) at donation	1.11	[1.07; 1.15]	**<0.001**
<40 years	Reference	​	​
40-60 years	4.794	[0.637; 36.098]	**0.128**
>60 years	6.62	[3.56; 12.31]	**<0.001**
Male gender	1.256	[0.688; 2.294]	0.458
BMI (kg/m^2^)	1.028	[0.958; 1.104]	0.441
BMI >30 kg/m^2^	1.707	[0.855; 3.409]	0.130
Active smoker	0.540	[0.236; 1.234]	0.144
Blood pressure parameters			
Mean arterial pressure (mmHg)	1.039	1.010; 1.069	**0.008**
Systolic BP (mmHg)	1.032	1.014; 1.051	**<0.001**
Diastolic BP (mmHg)	1.024	0.990; 1.059	0.175
24h SBP (mmHg)	1.033	1.009; 1.057	**0.008**
24h DBP (mmHg)	1.022	0.984; 1.062	**0.255**
Comorbidities			
Hypertension	2.073	1.130; 3.802	**0.002**
Prediabetes	1.890	0.631; 5.661	0.256
Hyperlipidemia	2.399	1.059; 5.437	**0.036**
Metabolic syndrome	1.150	0.333; 3.030	**0.799**
Thyroid disease	2.296	1.142; 4.617	**0.020**
Cardiovascular disease	1.47	0.50; 4.36	0.484
Malignant diseases	2.119	0.460; 9.759	0.335
COPD/Asthma/Chronic bronchitis	-	0.000; lnf	​
Medication use			
Antihypertensive medication	2.493	1.355; 4.586	**0.003**
Number of antihypertensives	1.474	1.073; 2.024	**0.017**
Lipid-lowering drugs	3.920	1.598; 9.617	**0.003**
Thyreoid medication	2.021	0.962; 4.246	0.063
Baseline laboratory			
Creatinine (mg/dL)	53.750	8.808; 327.982	**<0.001**
MDRD eGFR (mL/min/1.73 m^2^)	0.953	0.936; 0.970	**<0.001**
CKDepi eGFR (mL/min/1.73 m^2^)	0.924	0.903; 0.946	**<0.001**
Cockroft-gault eGFR (mL/min)	0.962	0.947; 0.977	**<0.001**
MAG3-clearance (mL/min/1.73m^2^)	0.992	0.984; 0.999	0.2500
Creatinine-clearance (mL/min/1.73m^2^)	0.985	0.972; 0.997	0.1600
Hemoglobin (g/dL)			
Cholesterol (mg/dL)	0.997	0.989; 1.005	0.432
Triglycerids (mg/dL)	0.998	0.993:1.003	0.484
LDL cholesterol	0.995	0.984; 1.021	0.964
HDL cholesterol (mg/dL)	1.000	0.978; 1.337	0.074
Blood glucose	1.012	0.993; 1.031	0.210
HBA1c (%)	2.017	0.863; 4.710	0.105
TSH (mU/mL)	1.149	0.987; 1.337	0.074
Urea (mg/dL)	1.022	0.983; 1.062	0.280
Urine albumine (mg/L)	1.020	0.927; 1.124	0.681
Urine protein (g/L)			
<0.03 g/L	Reference	​	​
0,03–0.15 g/L	0.956	0.523; 1.750	0.885
>0.15 g/L	0.729	0.092; 5.772	0.765
Long-term follow-up data			
Time since donation (months)	1.002	0.998; 1.006	0.401
Age at follow-up (years)	1.105	1.069; 1.006	**<0.001**
Comorbidities			
Hypertension	2.319	1.133; 4.746	**0.021**
Diabetes mellitus	0.768	0.179; 3.292	0.722
Hyperlipidemia	1.948	1.060; 3.578	**0.032**
Thyroid disease	1.087	0.527; 2.243	0.821
Cardiovascular disease	2.794	1.232; 6.336	**0.014**
Malignant diseases	1.381	0.597:3.195	0.451
Major cardiovascular events	0.996	0.228; 4.338	0.995

BMI, Body Mass Index; BP, blood pressure; CI, confidence interval; CKDepi eGFR, glomerular filtration rate estimated by Chronic Kidney Disease Epidemiology Collaboration equation; COPD, chronic obstructive pulmonary disease; GFR, glomerular filtration rate; HbA1c–glycated hemoglobin A1c; HDL, high-density lipoprotein; LDL, low-density lipoprotein; MDRD eGFR, glomerular filtration rate estimated by Modification of Diet in Renal Disease equation; TSH, thyroid-stimulating hormone; OR, odds ratio: p–significance p.

Bold values indicate statistical significance.

A lower baseline GFR as measured by MDRD, CKDepi, and Cockcroft-Gault formulas predicted a higher risk (all p < 0.001).

At long-term follow-up, older age continued to increase the risk by approximately 10.5% per year. Hypertension, hyperlipidemia, and malignant diseases were also significantly associated with increased risk.

**FIGURE 6:**
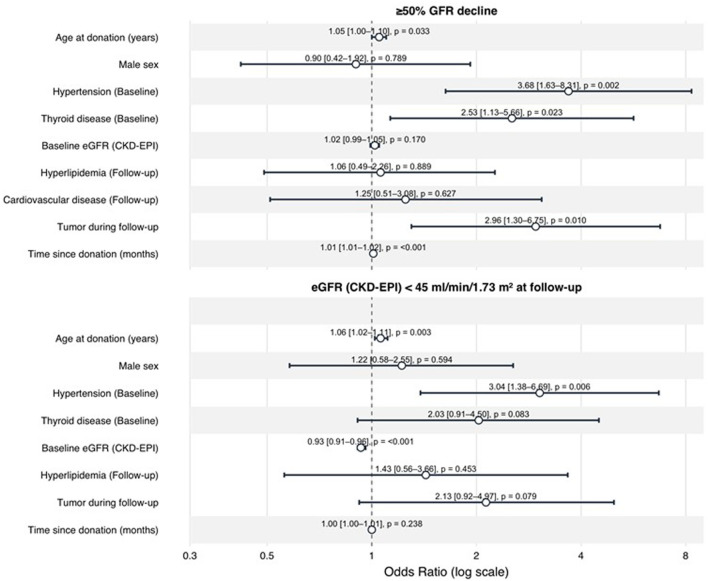
Multivariable logistic regression for adverse renal outcomes after living kidney donation. Forest plots display adjusted odds ratios (ORs) with 95% confidence intervals for predictors of (top) eGFR <45 mL/min/1.73 m^2^ at long-term follow up and (bottom) ≥50% eGFR decline from baseline at long-term follow-up.

In the multivariate analysis, age at donation, hypertension and baseline eGFR (OR 0.93), remained independent predictors (Figure 6).

### Determinants of Early Post-Donation eGFR-Decline

In multivariable linear regression analyses with absolute early eGFR change (ΔeGFR 0–12 months) as the dependent variable, baseline eGFR showed the strongest association with early decline (standardized β = −0.29, p < 0.001). Age at donation was independently associated with early decline but with a smaller effect size (standardized β = −0.16, p = 0.002). Hypertension showed a modest additional association (standardized β = −0.10, p = 0.023), whereas BMI and donation era were not independently associated with early eGFR change. When early eGFR change was expressed as a percentage of baseline function, age at donation and baseline eGFR showed comparable but smaller effect sizes (standardized β = −0.16 and −0.12, respectively), while BMI and donation era remained non-significant.

### Clinical Consequences and Preservation of Kidney Function

Severe renal impairment was rare, an eGFR <30 mL/min/1.73 m^2^ was observed in 1.2% and <15 mL/min/1.73 m^2^ in 0.3% of donors. Two patients required renal replacement therapy. Preservation of renal function (≤20% decline) was noted in 20.0% overall (32.4% in donors <40 years, 22.0% in donors 40–60 years, and 15.4% in donors >60 years).

MACE occurred in 4.3% (n = 27) of donors (Table 2), increasing with age (mean age at donation 55 years) and longer follow-up.

Twenty-one deaths occurred predominantly among older donors (mean age 73 ± 8.7 years at death), with malignancies accounting for 52.4%. Median time from donation to death was 14 years (IQR 10–19). Overall long-term survival was high across all age groups (Table 3).

### Longitudinal Changes in Laboratory Parameters

Glycemic control remained stable, although HbA1c levels increased slightly within the normal range from 5.45% ± 0.40% to 5.56% ± 0.58% (p < 0.001), mainly in donors aged 40–60 years.

Total cholesterol levels remained largely stable across the entire cohort, except for a small but significant increase in older donors (p = 0.0017), Table 2. Hemoglobin and hematocrit showed minimal changes, apart from a mild decline in hemoglobin among donors >60 years (p = 0.0092), Table 2.

### Exploratory Risk Stratification Based on Baseline Donor Characteristics

An exploratory risk stratification was performed to describe patterns of long-term renal outcomes according to baseline donor characteristics. Using baseline variables that were independently associated with an eGFR <45 mL/min/1.73 m^2^, including age (β = 0.062 per year), hypertension (β = 1.113), and baseline eGFR (β = −0.069 per mL/min/1.73 m^2^), a composite risk score was constructed to summarize their combined effects.

**FIGURE 7:**
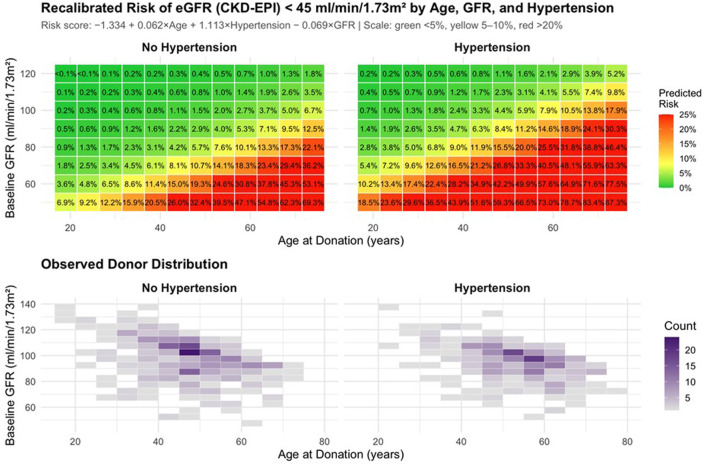
Predicted long-term risk of an eGFR <45 mL/min/1.73 m^2^ after living kidney donation, according to age at donation, baseline eGFR, and presence of hypertension. Top panels: Heatmaps display the predicted probability (%) of developing an eGFR <45 mL/min/1.73 m^2^ at long-term follow-up based on the recalibrated risk model (risk score = −1.334 + 0.062 × Age +1.113 × Hypertension – 0.069 × baseline eGFR). Colors indicate risk level (green <5%, yellow 5%–10%, orange 10%–20%, red >20%). Left: donors without baseline hypertension; right: donors with hypertension. Bottom panels: Observed distribution of donors by age and baseline eGFR, stratified by baseline hypertension status; shading reflects donor density.

**FIGURE 8:**
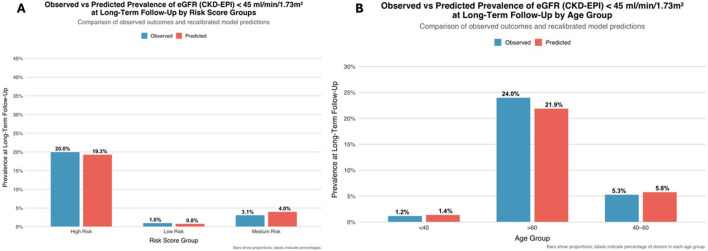
**(A)** Observed versus predicted prevalence of CKDepi eGFR <45 mL/min/1.73 m^2^ by risk-score groups. **(B)** Observed versus predicted prevalence of CKDepi eGFR <45 mL/min/1.73 m^2^ by age group. **(A)** Observed (blue) and predicted (orange) prevalence across low-, medium-, and high-risk tertiles. Predicted and observed rates (0.8% vs. 1.0%; 4.0% vs. 3.1%; 19.3% vs. 20.0%) confirmed accurate model calibration. **(B)** Model calibration across donor age categories (<40, 40–60, >60 years). Observed and predicted prevalence were closely aligned (1.2% vs. 1.4%; 5.3% vs. 5.8%; 24.0% vs. 21.9%, respectively).

A two-dimensional risk map illustrated the joint association of age, hypertension, and baseline eGFR with the prevalence of eGFR <45 mL/min/1.73 m^2^, showing a progressive increase with advancing age and lower baseline kidney function, particularly among hypertensive donors (Figure 7). When grouped into tertiles, the observed prevalence of eGFR <45 mL/min/1.73 m^2^ increased from 1.0% in the lowest to 20.0% in the highest risk category (Figure 8A). Across age strata, predicted and observed prevalences were closely aligned (<40 years: 1.4% vs. 1.2%; 40–60 years: 5.8% vs. 5.3%; >60 years: 21.9% vs. 24.0%; Figure 7B).

**FIGURE 9:**
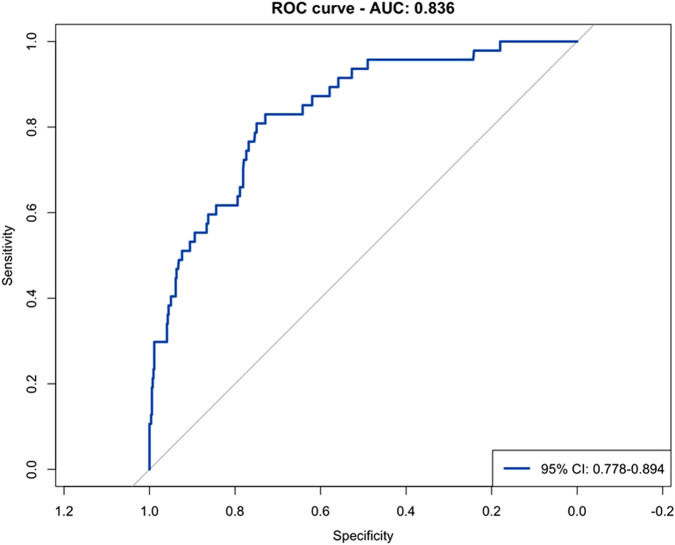
Receiver operating characteristic (ROC) curve for prediction of CKDepi eGFR <45 mL/min/1.73 m^2^. Observed (blue) and predicted (orange) prevalence across low-, medium-, and high-risk tertiles. Predicted and observed rates (0.8% vs. 1.0%; 4.0% vs. 3.1%; 19.3% vs. 20.0%) confirmed accurate model calibration.

Model discrimination was quantified for descriptive purposes and showed an AUC of 0.84 (95% CI 0.78–0.89; Figure 9).

### Cardiovascular Disease in Older Donors

Cardiovascular disease at baseline was uncommon overall but more frequent in older donors. Among donors >60 years, 19 of 115 (16.5%) had cardiovascular disease at baseline. Baseline kidney function was comparable between donors with and without cardiovascular disease. Long-term renal outcomes were also similar: eGFR <45 mL/min/1.73 m^2^ occurred in 21.1% versus 21.9%, and a ≥50% decline in eGFR in 23.5% versus 13.8%, respectively. Major adverse cardiovascular events occurred in 10.5% versus 6.3%.

### Outcome of Donors Aged ≥70 years

Among donors aged ≥70 years (mean 72, range 70–78 years), 55% were male. Most donated to their children (45%) or partners (35%). The mean follow-up period was 10.1 ± 2.5 years (range 7.1–14.7). Baseline eGFR (CKDepi) was 84.7 ± 9.0 mL/min/1.73 m^2^, and 75% had at least one comorbidity, most commonly hypertension (65%), hyperlipidemia (30%), and cardiovascular disease (30%), Table 6.

**TABLE 6:** Baseline characteristics and long-term outcome of living kidney donors aged ≥70 years (n = 21).

Parameter	Baseline	Last follow-up	Significance baseline – last follow up
Hypertension	65.0	85.0	0.125
Diabetes mellitus	0.0	5.0	0.004
Prediabetes	20.0	35.0	0.480
Hyperlipidemia	30.0	50.0	0.125
Cardiovascular disease	30.0	40.0	0.500
Malignancy	0.0	10.0	0.487
Thyroid disease	25.0	25.0	-
COPD/Asthma/Chronic bronchitis	0.0	0.0	​
Impaired mental health	5.0	5.0	0.997
Family history of kidney disease	40.0	40.0	0.998
CKDepi GFR (mean +/- SD)	84.7 ± 9.0	53.6 ± 14.7	**<0.01**
Drop of 50% eGFR	-	18.75	​
eGFR <45 mL/min/1.73 m^2^	0.0	25.0	-
eGFR <30 mL/min/1.73 m^2^	0.0	0.0	-
Renal replacement therapy	0.0	0.0	-
Death	-	4.8	-
Composite MACE	0.0	15.0	0.25

Continuous variables are presented as mean ± standard deviation (SD), and group comparisons were performed using the non-parametric Kruskal-Wallis test. Categorical variables are shown as percentages, and were compared using the Chi-square test or Fisher’s exact test, as appropriate. A p-value less than 0.05 was considered statistically significant.

Abbreviations: CKDepi GFR, glomerular filtration rate estimated by Chronic Kidney Disease Epidemiology Collaboration equation; COPD, chronic obstructive pulmonary disease; eGFR, estimated glomerular filtration rate; MACE, major adverse cardiac event; SD, standard deviation.

Bold values indicate statistical significance.

After donation, the mean eGFR decreased by 37.7% and subsequently remained stable (year 1: 54.5 ± 20.0 mL/min/1.73 m^2^ at year one, last follow-up: 53.6 ± 14.7 mL/min/1.73 m^2^; mean age 82 years). A ≥50% decline in eGFR occurred in 18.8%, and 25% had eGFR <45 mL/min/1.73 m^2^, but none dropped below 30 mL/min/1.73 m^2^ or required renal replacement therapy.

MACE occurred in 15%, while diabetes, malignancy, and psychiatric disorders were rare. One donor died from prostate cancer during long-term follow-up.

## Discussion

In this prospective caucasian cohort with long-term follow-up, living kidney donation demonstrated favorable clinical outcomes across a broad age spectrum. The mean donor age of 51 ± 11 years was 5–10 years higher than in most international cohorts (e.g., US: 41 ± 12 years; New Zealand: median 44 years) [21–23]. The donor population in this study closely resembled contemporary European cohorts, including the SoLKiD German National Registry (GNR) [24] and other European cohorts [7, 24, 25].

Importantly. living kidney donors do not represent a uniformly healthy cohort, but rather reflect an aging population and the gradual expansion of donor eligibility criteria driven by long waiting time for a brain-death donor due to donor shortage in Germany. Overweight and obesity were widespread, affecting 40.5% of donors and cardiometabolic risk factors were already prevalent at baseline. Hypertension was present in nearly half of donors, hyperlipidemia and prediabetes were less frequent, and approximately one-quarter of donors were active smokers, particularly among younger individuals. In contrast, established cardiovascular disease, malignancy, and mental health impairment were rare at the time of donation.

Women constituted 62.5% of donors, particularly in the youngest age group (71%), likely reflecting sociocultural factors and clinical considerations. This requires particular attention as donation by women of child-bearing age bears special risks due to potential pregnancy-related complications following nephrectomy [26, 27]. Sex was not associated with long-term kidney function decline in this cohort, consistent with several prior studies, although sex-specific effects remain inconsistently reported in the literature [10, 11, 28–30]. Nearly two-thirds of donors were biologically related to their recipients, which may modestly increase long-term renal risk through shared genetic or environmental factors [10, 11, 31, 32].

A key finding is the clear separation between early post-donation kidney function changes and long-term trajectories. As expected, eGFR declined by approximately 26% within the first year after donation, consistent with physiological adaptation to nephrectomy. The magnitude of early eGFR decline during the first year after donation was largely driven by baseline eGFR and, to a lesser extent, by age and preexisting hypertension. Donors with higher baseline eGFR experienced greater absolute early declines, consistent with a physiological ceiling effect of renal adaptation [33, 34]. In stratified analyses, donors with obesity alone showed slightly higher baseline eGFR values and comparable long-term trajectories. This likely reflects selection effects and higher baseline renal reserve, as obese individuals are accepted for donation only after careful screening, and higher baseline eGFR may partly reflect adaptive hyperfiltration.

After this initial adaptive phase, kidney function remained stable over long-term follow-up across all age groups. This observation aligns with prior work describing rapid compensatory hyperfiltration within the first months to years after donation, followed by a plateau phase without evidence of progressive decline in most donors [35]. Given the comparatively high donor age in many European countries [7, 15], our analyses extend prior work by disentangling age-related differences in achieved post-donation kidney function from age-related differences in the rate of kidney function change over time. Linear mixed-effects models demonstrated that older age at donation was associated with lower eGFR values throughout follow-up, whereas the slope of eGFR change did not differ significantly by age. Thus, age primarily influenced the level of kidney function prior to donation and immediately after donation rather than accelerating post-donation decline. The average decline of 35.1% was comparable to previous studies reporting a post-donation GFR decrease of 25%–40% with a similar or only marginally higher long-term risk of ESRD compared to the general population [4, 28, 32]. Even among donors aged ≥70 years, severe renal impairment was rare, and no donor progressed to ESRD.

Long-term renal impairment, as assessed by two primary endpoints, followed a clear age-related gradient but remained clinically reassuring in terms of severe outcomes. The primary endpoint of an eGFR <45 mL/min/1.73 m^2^ - was observed in 8.0% overall, 20.4% in donors age >60 years. Severe renal impairment was rare, with eGFR <30 mL/min/1.73 m^2^ in 1.2% and eGFR <15 mL/min/1.73 m^2^ in 0.3% of donors. The second primary endpoint - aGFR decline ≥50% at long-term - occurred in 6.8% overall and increased with age (4.8% < 40 years, 5.3% 40–60 years, 14.4% > 60 years). Beyond age, renal risk was highly heterogeneous and strongly modified by baseline risk profile. In younger donors with favorable risk profiles (no hypertension, BMI <25 kg/m^2^), the prevalence of reduced eGFR was negligible, but increased to 4.5%–10.6% in young donors with hypertension or obesity. In older donors, risk likewise varied considerably, ranging from low levels in donors without risk factors up to 45.5% in donors with hypertension and a BMI ≥30 kg/m^2^. These findings underscore that chronological age alone is insufficient for individualized risk assessment and that long-term renal outcomes after donation are shaped by the interaction between baseline renal reserve and comorbidity burden—such that younger donors with adverse metabolic profiles may experience less favorable outcomes than some older but otherwise healthy donors.

Cardiovascular risk factors were also common in younger donors. Smoking was more prevalent among younger donors, whereas hypertension affected more than 65% of donors older than 60 years at follow-up. Although cardiovascular disease was rare at donation, its prevalence increased over time, especially among older donors. At time of donation, only 0.9% had relevant cardiovascular disease, increasing to 6.6% overall and 16.5% among donors over 60 years. Similarly, Colucci et al. reported only 3.2% new-onset cardiovascular disease after 10 years of follow-up [25]. A Norwegian registry study observed a modestly increased risk of ischemic heart disease in donors (3.5%) compared with controls (1.7%) [36], while other large studies found no significant difference in cardiovascular morbidity and mortality between donors and healthy non-donors [37, 38]. Overall, the available evidence suggests that living kidney donation, when donors are carefully selected and monitored, is not associated with a substantial excess cardiovascular risk. The observed rise in modifiable risk factors such as hypertension and hyperlipidemia over time underscores the importance of structured long-term care, including lifestyle counseling and optimized blood-pressure management.

Longitudinal follow-up highlighted additional clinically relevant aspects. Younger age does not protect against the development of new comorbidities, including mental health impairment, which remains an often underrecognized aspect of donor follow-up. Psychological screening and support should be integral components of long-term donor care, even for individuals initially classified as low risk. Similarly, the rise in malignancy incidence, particularly among older donors, appears age-related rather than donation-related but supports the need for age-appropriate cancer surveillance.

Finally, the present study explores whether data-informed approaches may help to characterize heterogenity of long-term renal risk after living kidney donation. While earlier models, such as that by Grams et al., estimated lifetime ESRD risk in potential donors [31], the present analyses addressed post-donation renal outcomes within a well-characterized donor cohort. Exploratory analyses suggested that a limited set of routinely available baseline clinical parameters - particularly baseline eGFR and hypertension status, togethter with age - captured a substantial proportion of the variation in long-term renal outcomes. Although not intended as a predictive tool, this approach illustrates how readily available clinical information may support more individualized counseling and risk-adapted follow-up strategies. Given that the HeiKiD approach was derived from a predominantly Caucasian European donor cohort, its applicability is likely most relevant to similar donor populations. Such approaches may complement clinical judgment but require external validation before broader application.

This study has limitations. The cohort represents caucasian donors mainly from Germany; however, donor characteristics were comparable to national and other European cohorts [7, 24, 25], suggesting reasonable generalizability within similar healthcare settings. In addition, the absence of a non-donor control group precludes direct estimation of donation-attributable risk. Accordingly, the observed outcomes describe post-donation trajectories within the donor population rather than comparative risk relative to non-donors.

In conclusion, this long-term prospective cohort demonstrates stable kidney function after the expected early adaptive decline following living kidney donation, even in an aging donor population. Clinically relevant renal impairment increased with age, yet severe renal impairment and renal replacement therapy were rare. Long-term outcomes were not driven by chronological age alone but reflected the combined influence of baseline renal reserve, hypertension, and comorbidity burden. Using a small number of routinely available clinical parameters, long-term renal risk could be meaningfully characterized, supporting individualized donor counseling and risk-adapted lifelong follow-up.

## Data Availability

The datasets presented in this article are not readily available because partial restrictions apply to the availability of these data, which were used under license for the current study, and so are not publicly available. Requests to access the datasets should be directed to claudia.sommerer@med.uni-heidelberg.de.
